# Differential intracellular calcium influx, nitric oxide production, ICAM-1 and IL8 expression in primary bovine endothelial cells exposed to nonesterified fatty acids

**DOI:** 10.1186/s12917-016-0654-3

**Published:** 2016-02-25

**Authors:** Anitsi Loaiza, María D. Carretta, Anja Taubert, Carlos Hermosilla, María A. Hidalgo, Rafael A. Burgos

**Affiliations:** Instituto de Farmacología, Facultad de Ciencias Veterinarias, Universidad Austral de Chile, Valdivia, Chile; Institute of Parasitology, Faculty of Veterinary Medicine, Justus Liebig University Giessen, Giessen, Germany

**Keywords:** Nonesterified fatty acid, Primary bovine endothelial cell, Interleukin 8, Intercellular adhesion molecule 1, Calcium flux

## Abstract

**Background:**

Nonesterified fatty acids (NEFAs) are involved in proinflammatory processes in cattle, including in the increased expression of adhesion molecules in endothelial cells. However, the mechanisms underlying these effects are still unknown. The aim of this study was to assess the effects of NEFAs on the intracellular calcium (Ca^2+^_i_) influx, nitric oxide production, and ICAM-1 and IL-8 expression in primary bovine umbilical vein endothelial cells (BUVECs).

**Results:**

Myristic (MA), palmitic (PA), stearic (SA), oleic (OA) and linoleic acid (LA) rapidly increased Ca^2+^_i_. The calcium response to all tested NEFAs showed an extracellular calcium dependence and only the LA response was significantly inhibited until the intracellular calcium was chelated. The EC_50_ values for MA and LA were 125 μM and 37 μM, respectively, and the MA and LA effects were dependent on calcium release from the endoplasmic reticulum stores and on the L-type calcium channels. Only the calcium response to MA was significantly reduced by GW1100, a selective G-protein-coupled free fatty acid receptor (GPR40) antagonist. We also detected a functional FFAR1/GPR40 protein in BUVECs by using western blotting and the FFAR1/GPR40 agonist TAK-875. Only LA increased the cellular nitric oxide levels in a calcium-dependent manner. LA stimulation but not MA stimulation increased ICAM-1 and IL-8-expression in BUVECs. This effect was inhibited by GW1100, an antagonist of FFAR1/GPR40, but not by U-73122, a phospholipase C inhibitor.

**Conclusions:**

These findings strongly suggest that each individual NEFA stimulates endothelial cells in a different way, with clearly different effects on intracellular calcium mobilization, NO production, and IL-8 and ICAM-1 expression in primary BUVECs. These findings not only extend our understanding of NEFA-mediated diseases in ruminants, but also provide new insight into the different molecular mechanisms involved during endothelial cell activation by NEFAs.

**Electronic supplementary material:**

The online version of this article (doi:10.1186/s12917-016-0654-3) contains supplementary material, which is available to authorized users.

## Background

The levels of nonesterified fatty acids (NEFAs) in bovine plasma increase during transition periods, such as after calving and during early lactation [1]. During these stages, a negative energy balance is generated, which is mainly characterized by an increased release of NEFAs from adipose tissues into the plasma [2]. Elevated plasma NEFA concentrations have recently been related to the incidence of metabolic and infectious bovine diseases, including fatty hepatitis, metabolic ketosis, mastitis, and endometritis, which cause high economic losses to the cattle industry worldwide [2, 3]. Therefore, NEFAs directly affect tissue/organ functions, and even more importantly, indirectly affect the bovine innate immune system, as previously reported [4].

Endothelial cells are highly immunoreactive cells that synthesize a broad spectrum of chemokines, cytokines, and adhesion molecules, and therefore play an active part in early proinflammatory reactions [5]. Consistent with this, the exposure of primary bovine aortic endothelial cells (BAECs) to a mixture of NEFAs for 24 h altered their membrane phospholipid profile, increasing the stearic acid concentration and reducing the polyunsaturated fatty acid concentrations [6]. In the same study, 0.75 mM NEFA increased the expression of the proinflammatory cytokines interleukin 6 (IL-6) and IL-8, and the adhesion molecules intercellular adhesion molecule 1 (ICAM-1) and vascular cell adhesion molecule 1 (VCAM-1) in BAECs [6], which interact with other immunocompetent cells, such as neutrophils. Although the effects of NEFAs on bovine leukocytes and endothelial cells have been examined previously [1, 7, 8], the exact molecular mechanisms are still poorly understood.

It has long been accepted that the hydrophobic nature of lipids allows them to activate cells after they enter them, e.g., through membrane transport mechanisms [9, 10]. However, a G-protein-coupled free fatty acid receptor known as GPR40 or free fatty acid receptor 1 (FFAR1) has recently been described [11]. This novel free fatty acid receptor recognizes saturated and unsaturated medium- and long-chain fatty acids, and the human, rat, and mouse isoforms of FFAR1/GPR40 have been described [11, 12]. Recently, the effects of FFAR1/GPR40 agonists, such as oleic acid (OA) and linoleic acid (LA), have been demonstrated in epithelial mammary cells [13] and neutrophils [14, 15]. Consequently, primary bovine tissues are thought to be susceptible to NEFA exposure primarily *via* the corresponding bovine free fatty acid receptor 1 (bFFA1R/bGPR40). Based on this evidence, we evaluated whether different types of NEFAs can rapidly modify the intracellular calcium response in primary bovine endothelial cells exposed to single fatty acids and to study in more detail the molecular mechanisms involved in this endothelial activation.

## Results

### Acute treatment with NEFAs does not affect the viability of primary bovine umbilical vein endothelial cells (BUVECs)

Cells exposed to 300 μM LA, palmitic acid (PA), OA, myristic acid (MA), or stearic acid (SA) showed no significant difference in the propidium iodide signal for 15 min when compared with untreated cells (basal condition) (see Additional file 1). Similar results were observed in BUVECs exposed to 1 % vehicle (DMSO or ethanol) for the same period. Therefore, exposure to 300 μM of each fatty acid did not increase BUVEC death any more than Triton X-100 treatment, used as the positive control. We demonstrated that 0.3 mM EGTA, 50 μM BAPTA-AM or each NEFA plus EGTA or BAPTA did not affect the viability of BUVECs (see Additional file 1). These results clearly demonstrate that these fatty acid concentrations and exposure time have no toxic effects on BUVECs.

### NEFAs increase the intracellular calcium influx in BUVECs

**Fig. 1:**
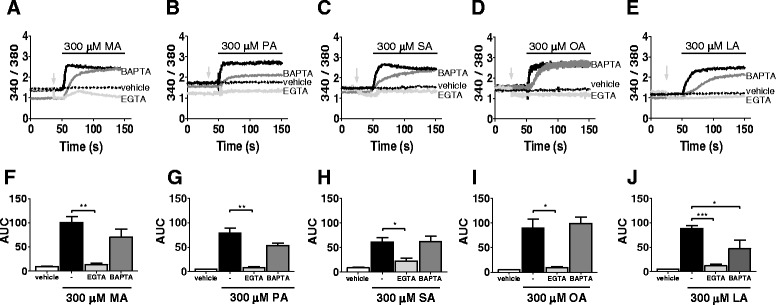
Intracellular calcium increases caused by NEFAs are dependent on extracellular calcium. **a**–**e** Time courses of representative Fura-2 ratio signals in at least three assays, caused by 300 μM of each NEFA in BUVEC cells. Each NEFA was added at 50 s during a 100 s period (*black line*). Suspended cells were exposed to each NEFA in HBSS, without or with pretreatment with 50 μM BAPTA-AM or calcium-free HBSS/0.3 mM EGTA (*black, dark grey,* and *light grey traces*, respectively). Before fatty acid exposure, EGTA was added at the *light grey arrow*. BUVECs were also exposed to only to the vehicle control at 50 s (*dotted traces*). Ethanol (1 %) was used as the vehicle for MA and SA. DMSO (1 %) was used as the vehicle for PA, OA, and LA. **f**–**j** AUCs for NEFAs in HBS buffer (*black bars*), calcium-free buffer with 0.3 mM EGTA (*light grey bars*), or after BAPTA treatment (*dark grey bars*). AUC of vehicle alone, 1 % ethanol or 1 % DMSO (white bars). The data are expressed as the means ± SEM of at least three experiments. *p < 0.05, **p < 0.01, ***p < 0.005

The intracellular calcium response in primary bovine endothelial cells was evaluated in BUVECs exposed acutely to different NEFAs for 100 s. The calcium signal increased quickly after stimulation with 300 μM MA, PA, SA, or OA, with similar kinetics, and the intracellular calcium levels reached a new steady state (Fig. 1a–d, black traces). In contrast, LA caused a slow but constant increase in intracellular calcium (Fig. 1e). To identify the roles of intracellular and extracellular calcium, we used the well-known calcium-chelating agents BAPTA-AM and EGTA. Incubation with BAPTA-AM reduced the slope of calcium increase by more than 50 % in all cases, except for LA (see Additional file 2). However, this did not affect the area under the curve (AUC) of calcium flux (Fig. 1f–i). Moreover, the BAPTA-AM treatment significantly reduced the area under the curve (AUC) only in cells previously exposed to LA (Fig. 1j). The latter suggests that the increase in calcium induced by LA is dependent on intracellular and extracellular calcium mobilization. In our experiments, the NEFA-mediated increase in calcium was significantly inhibited in the presence of EGTA (as illustrated in Fig. 1a–e, light gray traces; Fig. 1f–j), suggesting that the increase in calcium in the whole experiment is mainly depended on calcium influx.

### Mechanisms of MA and LA effects on intracellular calcium release in BUVECs

**Fig. 2:**
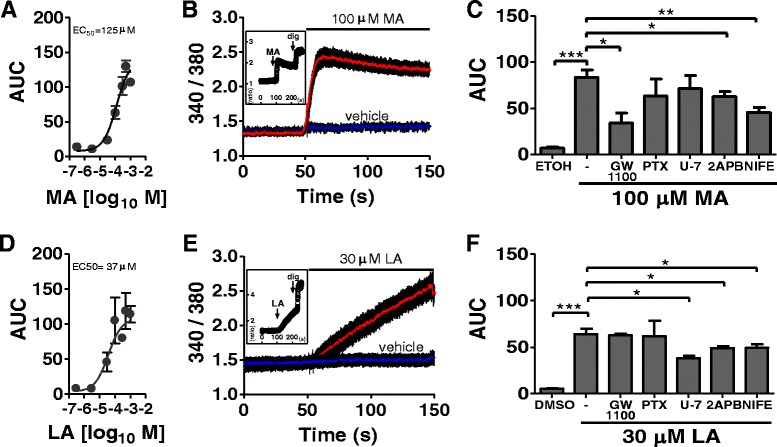
Signaling routes of the calcium responses caused by MA and LA in BUVECs. **a** and **d** Dose–response of calcium signaling induced by myristic acid (MA) or linoleic acid (LA). The area under the curve (AUC) calculated for the first 100 s of the Fura-2/AM time course. **b** and **e** Average time courses for the Fura-2 ratio in BUVECs exposed to 100 μM MA in ethanol or 30 μM LA in 1 % DMSO. Graph inset: whole time course of the Fura-2/AM ratio after exposure to 100 μM MA before the addition of 132 μM digitonin (dig) (representative assay). **c** and **f** Calcium response in cells triggered by 1 % ethanol or 1 % DMSO, 100 μM MA or 30 μM LA alone or after preincubation with 100 μM 2-APB, 10 μM GW1100, 4 μM U73122 (U7), or 20 μM nifedipine (NIFE) for 15 min. Cells were incubated for 16 h with 100 ng/ml pertussis toxin (PTX) before MA or LA supplementation. The data are expressed as the means ± SEM of at least three independent experiments. *p < 0.05, **p < 0.01, ***p < 0.005

We investigated the potential differential responses of BUVECs to MA and LA, exemplary for saturated and unsaturated NEFAs, respectively. To investigate the mechanism of the calcium increase, we first constructed dose–response curves of the calcium signal after stimulation with MA and LA, and obtained half-maximal effective concentrations (EC_50_) of 125 and 37 μM, respectively (Fig. 2a, d). We then used concentrations close to the EC_50_ values to assess the mechanisms involved in calcium flux. Upon stimulation with 100 μM MA, BUVECs showed a rapid increase in calcium, which decreased until a new steady state was reached after 30 s (Fig. 2b, inset). In contrast, stimulation with 30 μM LA caused a sustained increase in calcium within the first 100 s (see Fig. 2e, inset), and a steady state was observed after stimulation for 200 s (data not shown).

To evaluate whether FFAR1/GPR40 actively participates in the calcium increase induced by MA or LA, we used its antagonist GW1100, as previously reported [16]. The response produced by 100 μM MA was inhibited by 59 % by 10 μM GW1100 (see Fig. 2c). This was also observed when other saturated fatty acids were used in our in vitro bovine endothelial cell system, and GW1100 inhibited 66 and 63 % of the calcium response induced by 300 μM PA and SA, respectively (see Additional file 3). In contrast, the calcium influx response to 30 μM LA was not affected by GW1100 (Fig. 2f), and a similar response was observed when OA was used as the unsaturated fatty acid (see Additional file 3).

To assess the role of the G-protein Gα_i/o_, phospholipase C (PLC), and the inositol triphosphate (IP_3_) receptors/calcium-operated channels induced by MA and LA, we used pertussis toxin (PTX) [17], U73122 [18], and 2-APB [19–21], respectively. We demonstrated that preincubation with 100 ng/ml PTX for 16 h did not inhibit the calcium influx induced either by MA or LA fatty acids in BUVECs, which suggests that Gα_i/o_ does not participate in this type of response. The mobilization of calcium by LA was reduced by 4 μM U73122, but the effect of MA was not affected by treatment with the PLC inhibitor. 2-APB (100 μM) reduced both the MA- and LA-mediated increases in calcium by 25 and 23 %, respectively (see Fig. 2c and f).

There is some evidence that fatty acids can directly and indirectly activate the L-type calcium channels (LTCCs) in HEK293, COS-7 and rat insulinoma INS-1E cells in vitro [22, 23]. However, the participation of LTCCs in the calcium increase induced by MA or LA in BUVECs has not been evaluated. Therefore, we investigated whether these channels participate in the calcium increase induced by MA or LA stimulation. Our results show that the LTCC blocker nifedipine (20 μM) inhibited the increases in calcium induced by MA and LA by 45 and 22 %, respectively (Fig. 2c and f). These results suggest that LTCCs participate in the increases in calcium caused by saturated and unsaturated fatty acids in BUVECs.

### FFAR1/GPR40 expression and activity

**Fig. 3:**
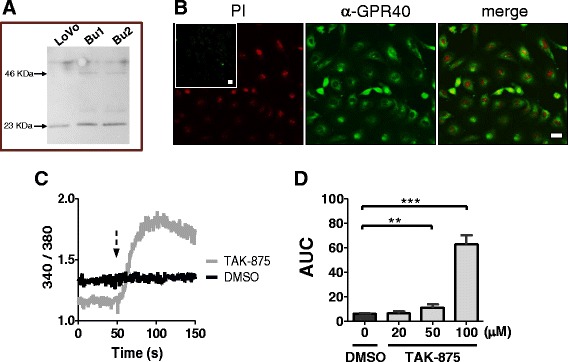
Protein expression and functional detection of FFAR1/GPR40 in BUVEC monolayers. **a** Western blot of bovine FFAR1/GPR40. Total protein extract from LoVo cells was used as the positive control for two BUVEC isolates. 50 μg of total protein was loaded onto a 6 % polyacrylamide gel. **b** FFAR1/GPR40 immunofluorescence. *Left image*, nuclear signals of cells incubated with propidium iodide (PI); inset image: only the secondary antibody was used, as the negative control, bar = 20 μm. *Middle image*, FFAR1/GPR40 detected with rabbit anti-GPR40 antiserum and an anti-rabbit IgG secondary antibody. *Right image*, nuclear signals and GPR40 images are superimposed, bar = 20 nm. **c** Representative time course of the Fura-2/AM ratio in BUVECs exposed to 1 % DMSO or 100 μM TAK-875 at the time indicated by the arrow. **d** Cells were exposed to DMSO or TAK-875. The area under the curve (AUC) was calculated during the first 100 s after exposure to the stimulus. The data are expressed as the means ± SEM of at least three independent experiments. **p < 0.01, ***p < 0.005

To examine FFAR1/GPR40 protein expression in BUVECs, we used western blotting and immunofluorescence assays directed against the conserved epitope of bovine GPR40, as described elsewhere [14]. An immunoblotting analysis of the total protein extracts of BUVECs showed a band with an apparent molecular size of 23-kDa, lower than expected but similar to that observed in LoVo cells, which were used as the positive control, as suggested by the manufacturer of the anti-GPR40 antibody (Fig. 3a). The same antibody was used for the immunofluorescence assays of BUVECs, which detect the internal region of human GPR40, and therefore the intracellular and plasma membrane distribution (Fig. 3b). To confirm that the FFAR1/GPR40 protein is in its active form in BUVECs, we also measured the calcium signal separately with two known FFAR1/GPR40 agonists, TAK-875 [24] and GW9508 [16]. TAK-875 increased the intracellular calcium in a dose-dependent manner (Fig. 3c, d), whereas GW9508 did not change the calcium signal at either low (10, 50 μM) or high concentrations (100 μM; data not shown).

### LA increases nitric oxide (NO) production

**Fig. 4:**
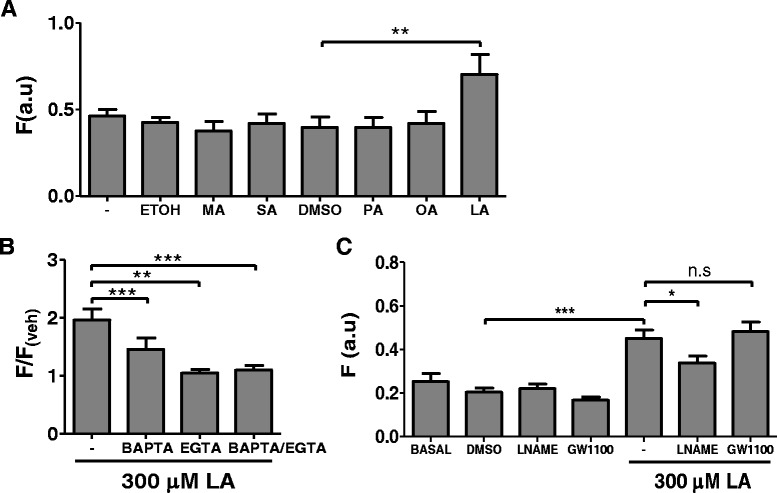
NEFA effects on nitric oxide production in BUVECs. **a** Basal DAF fluorescence (−), and after treatment with 0.1 % ethanol, 300 μM myristic acid (MA), 300 μM stearic acid (SA), 0.1 % DMSO (D), 300 μM palmitic acid (PA), 300 μM oleic acid (OA), or 300 μM linoleic acid (LA) for 5 min at 37 °C. **b** DAF fluorescent signal after incubation with 300 μM LA for 5 min, normalized to the signal without fatty acids. The cells were incubated with BAPTA, EGTA, or BAPTA + EGTA before treatment with LA. **c** Effect of L-NAME and GW1100 on the LA-induced increase in the DAF signal. Basal fluorescence. DAF fluorescence is expressed in arbitrary units (a.u.). Each bar represents the mean ± SEM of at least three independent experiments. *p < 0.05, **p < 0.01, ***p < 0.005

To assess whether NEFAs affect NO production in the endothelium, we exposed BUVECs to MA, SA, PA, OA, or LA and evaluated the cells for NO production using DAF-FM signals. Only LA significantly increased the DAF-FM fluorescence intensity in BUVEC monolayers. As illustrated in Fig. 4a, the DAF-FM signal increased approximately 1.5-fold in LA-exposed BUVECs compared with the signal in cells treated with vehicle (DMSO). The increase in NO induced by LA was reduced by 25 % when the cells were treated with 100 μM L-NAME, a potent endothelial nitric oxide synthase (eNOS) inhibitor (Fig. 4c). This indicates that LA activates eNOS in primary bovine endothelial cells. Treatment with BAPTA, EGTA, or BAPTA plus EGTA reduced the increase in NO induced by LA by 50, 91, and 86 %, respectively (Fig. 4b). In contrast, neither 4 μM U73122, 10 μM GW1100, 100 μM 2-APB, 20 μM nifedipine, nor 100 ng/ml PTX reduced the LA-mediated increase in NO (data not shown).

### LA but not MA increases ICAM-1 and IL-8 mRNA expression

**Fig. 5:**
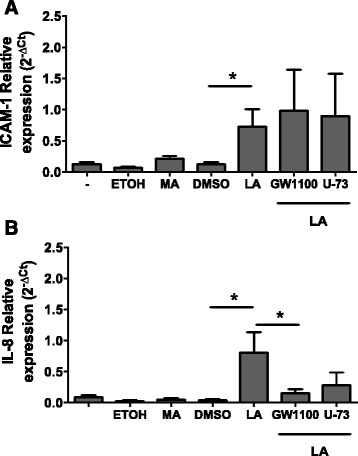
NEFA effects on ICAM-1 and IL-8 expression in BUVECs. Cells were treated with 0.1 % ethanol, 300 μM myristic acid (MA), 0.1 % DMSO, 300 μM oleic acid (OA), or 300 μM linoleic acid (LA) for 3 h and their RNAs were then isolated. Some cells were treated with 10 μM GW1100 or 4 μM U73122 (U-73) for 30 min before the LA treatment. The expression of ICAM-1 and IL-8 was analyzed with real-time PCR using specific primers. Relative abundance = 2^–ΔCt^, where ΔCt = cycle treshold ICAM-1 or Ct IL-8 minus cycle treshold SDHA. The data are expressed as the means ± SEM of three experiments. *p < 0.05

Because only LA effectively increased eNOS activity in BUVECs, we tested whether LA also affects ICAM-1 or IL-8 expression. Only LA induced the expression of ICAM-1 and IL-8 mRNAs in BUVECs, and LA-induced IL-8 expression was significantly inhibited by GW1100. However, the expression of ICAM-1 was not reduced by treatment with the FFAR1/GPR40 antagonist (Fig. 5).

## Discussion

The NEFA levels in blood plasma are particularly elevated during the transition period in dairy cattle, when lipids are strongly mobilized from the adipose tissues to the bloodstream, with plasma concentrations increasing from the basal level of 0.1 mM to 0.7–2 mM [1, 25–27]. Until now, the involvement of G-coupled fatty acid receptors, such as FFAR1/GPR40, in the NEFA-mediated calcium response in primary endothelial cells has been unclear. This study has demonstrated that single NEFAs alone, at low concentrations of 0.3 mM, increase the intracellular calcium response in primary bovine endothelial cells, inducing different molecular pathways involved in the endothelial cell activation process. In naturally occurring acute systemic coliform mastitis linoleic acid and oleic acid were the most abundant NEFA, suggesting that specific fatty acids can be involved in inflammation and innate immunity, and to be relevant as risk factors during the transition period [28].

Different studies have reported the potency with which MA and LA bind to human FFAR1/GPR40 in transfected CHO and HEK293 cells by measuring the calcium signal. For MA, a EC_50_ value has been described in these cells, close to 7 μM, but for LA, the EC_50_ values ranged from 1.8 to 6.6 μM [11, 12]. In contrast, the EC_50_ values for MA and LA were 30.3 and 38.4 μM, respectively, in a reporter cell line based on HeLa cells transfected with hGPR40 [29]. These results are quite similar to those observed in the present study, especially regarding the stimulatory potency of LA. Because many of the effects of NEFAs on primary bovine endothelial cells could be attributed to bGPR40, including increased intracellular calcium influx, the presence of this specific NEFA receptor was assessed in BUVECs in this study. We detected FFAR1/GPR40 expression in BUVECs, and also evaluated its effect on the calcium flux using the agonist TAK-875 in our in vitro primary bovine endothelial cell system. There is some evidence that FFAR1/GPR40 is expressed in bovine cells and bovine FFAR1/GPR40 has been shown to share 84 % nucleotide sequence identity with the human isoform [13, 14]. Previous studies have examined the expression of GPR40 in endothelial cells from monkey brain tissues [30], neuromicrovascular cells from rodent tissues [31], and human cardiac endothelial cells [32]. To the best of our knowledge, this work is the first to report the expression of GPR40 in primary bovine endothelial cells.

Our data also suggest that the MA-mediated increase in calcium occurs when MA binds to bGPR40, because it was clearly inhibited by GW1100, a potent FFAR1/GPR40 antagonist. In contrast, the increase in calcium induced by LA does not involve this receptor, according to our antagonist experiments. We also observed that IP_3_ receptors/calcium-operated channels and LTCCs seem to participate in the cellular responses to MA and LA, respectively. Others have shown, that OA increased the calcium signal in HEK 293 cells transiently transfected with the α1 subunits of the LTCCs [23]. The pathway of calcium entry *via* the LTCCs induced by NEFAs is evidently cell-type dependent. LTCCs were also associated with the increase in calcium mediated by FFAR1/GPR40 activation that was induced by the saturated fatty acid PA in rat β-cell line INS-1E [22], by the unsaturated fatty acid OA in islet β-cells from rats [33], and by LA in primary cultured chicken hepatocytes [34]. Our examination of the cellular calcium flux suggests that MA, but not LA, recognizes FFAR1/GPR40 in BUVECs. The *CACNA2D1* gene encodes the alpha 2/delta subunit which is an accessory part of the voltage-gated calcium channel family (e.g., LTCC) and considered to be an important candidate protein influencing bovine mastitis [35]. Although many publications have reported LTCC activity in bovine immune cells, there is not yet any evidence that these channels are expressed in bovine endothelial cells. In the present study, nifedipine affected BUVECs, suggesting the participation of LTCCs. It has also been recently demonstrated that the increase in calcium caused by endothelin-1 is LTCC-dependent in cultured bovine corneal endothelial cells [36].

We also evaluated the NO response in NEFA-exposed bovine endothelium, because NO is a well-known indicator of inflammation [37]. All NEFAs were evaluated, but only LA increased NO in the endothelial cells. This response was dependent on both calcium and eNOS. There is evidence that eNOS activity is dependent on Ca^2+^/calmodulin [38], and it is probable that the increase in NO is a secondary effect after the interaction of eNOS with Ca^+2^/calmodulin. Both NO and eNOS actively participate in the regulation of vascular tone and are therefore important intracellular enzymes, especially in microvascular hyperpermeability [39]. There is some evidence that increased levels of NO in endothelial cells produce proinflammatory profiles, which might lead to metabolic diseases. LA induces reactive oxygen species (ROS) and the overproduction of NO after an increase in calcium, causing peroxynitration in endothelial cells [40]. NF-κB, a crucial transcription factor, is then activated by peroxynitrite, a potent oxidative agent, inducing the expression of adhesion molecules [40] and chemokines, such as IL-8 [41]. We also demonstrated that LA, but not MA, induces ICAM-1- and IL-8-expression, suggesting an active role for NO in this response. In bovine endothelial cell cultures, high concentrations of total NEFAs might increase ICAM-1 mRNA and protein expression [6]. The LA-induced adhesion molecules and chemokines may participate in leukocyte-endothelial cell interactions, facilitating transendothelial migration [42], and may play a role in the onset of a proinflammatory process during the transition period in dairy cattle. Massive lipid mobilization leading to an increase in plasma NEFAs is observed in cows around parturition, causing an increased incidence of metabolic and infectious diseases, including laminitis, endometritis, and mastitis [1–4]. The exposure of bovine endothelial cells to NEFAs increases the expression of the proinflammatory cytokines IL-6 and IL-8 and the adhesion molecules ICAM-1 and VCAM-1 [6]. It has also been postulated that the excessive accumulation of NEFAs during chronic inflammatory responses might increase TNFα, IL-6, and IL-1β expression by activating NF-κB in adipocytes and macrophages. These cytokines are thought to link the metabolic state and the inflammatory response, usually in the postpartum period within the uteri of cows [3, 4].

There is evidence that endothelial calcium participates in the transendothelial lymphocyte migration caused by ICAM-1 [43]. Moreover, an intracellular calcium increase has been related with ICAM-1 expression in HUVEC [44]. However, in BUVECs, intracellular calcium increase could be not directly associated with the expression of ICAM-1 or IL-8. Consistently with these assumption MA increased calcium flux was not sufficient to increase the expression of these molecules and the inhibition of PLC did not reduce the expression of ICAM-1 and IL-8 induced by LA. Our data also demonstrate that the individual NEFAs have different effects on endothelial cell activity and diverse mechanisms of action. LA induces the expression of ICAM-1 independently of FFAR1/GPR40 or PLC activity, although the expression of IL-8 is associated with the activation of FFAR1/GPR40 in BUVECs. In contrast, our results showed that NO was induced by LA, in a rather G-protein-coupled-receptor-independent process. There is some evidence that these receptors need not bind to a G-protein in the presence of high agonist concentrations, which thereby alternatively activate the ERK/MAPK pathway [45]. It has been suggested that LA activates the phosphoinositide 3-kinase, ERK1/2, and p-38 MAPK pathways in vascular endothelial cells [46]. In our laboratory, we have recently shown that the increase in NO caused by LA is p38-dependent (unpublished data). For this reason, it is possible that LA activates FFAR1/GPR40 or another receptor in a G-protein-independent manner. For instance, we cannot exclude the possibility that the GPR120 receptor recognizes LA by another mechanism [16, 47], or that LA is recognized by GPR84, a medium-chain fatty acid receptor [48], or CD36, a fatty acid transporter protein [49, 50]. Only CD36 expression has been demonstrated in endothelial cells [51]. It has also been proposed that LA mobilizes intracellular calcium *via* an LA metabolite that induces intracellular mitochondrial calcium fluxes [52].

## Conclusions

We have demonstrated for the first time in primary bovine endothelial cells that GPR40 is involved in the increase in calcium induced by MA, and conversely, that the LA calcium flux is not associated with this receptor. We have also demonstrated the participation of IP_3_ receptors/calcium-operated channels and LTCCs in the cellular responses to MA and LA. We observed FFAR1/GPR40 expression in the bovine endothelium and confirmed its activity using its agonist, TAK-875. Only LA increased NO levels, and this was independent of FFAR1/GPR40. Only LA induced the expression of ICAM-1 and IL-8, and this phenomenon is a FFAR1/GPR40- dependent cellular process. These results indicate that the responses of endothelial cells to individual NEFAs are highly variable. Recently, it has been demonstrated that there is an association of LA enhancement in plasma and milk in naturally occurring acute systemic coliform mastitis [52]. Therefore, the increase in total NEFAs that is described as risk factor for the onset of proinflammatory process that occur during transition periods in cattle, should be revised carefully in the future. Further studies are required to determine whether other individual NEFAs participate in the bovine proinflammatory responses in vivo during the transition period and in other NEFA-dependent disorders in cattle.

## Methods

### Fatty acids

We used those fatty acids that have shown changes during transition period [1]. Myristic and stearic acids were purchased from Sigma Chemical Co. (St. Louis, MO, USA) and suspended in 100 % analytical-grade ethanol (Merck, Germany). Palmitic, oleic, and linoleic acids (Sigma Chemical Co. USA) were prepared in 100 % DMSO (Sigma Chemical Co. USA). Stocks of single fatty acids were prepared at concentrations of 300 mM, protected from light, and stored at −20 °C. To estimate the calcium dose responses, the fatty acids were diluted to the indicated concentrations, with the vehicle maintained at 1 % DMSO or 1 % ethanol to avoid a possible dilution effect of vehicles. Because 300 μM concentrations of all fatty acids were used in the NO experiments, 0.1 % DMSO or 0.1 % ethanol were used as the vehicles. Neither the DMSO nor ethanol concentration used for the calcium and NO assays affected the viability of BUVECs during period of exposure. The working stocks of fatty acids were maintained at 37 °C and were protected from light before to use.

### Cell culture

Primary BUVECs were isolated from bovine umbilical cords according to Hermosilla et al. [53]. All bovine umbilical cords were obtained as part of natural parturition procedures at the Clinic for Obstetrics and Gynaecology and Andrology of Large and Small animals, Faculty of Veterinary Medicine, Justus Liebig University Giessen, Germany therefore no intervention was made or ethic approval was requested.

Briefly, the umbilical veins were isolated from the umbilical cords of calves born by cesarean section and kept at 4 °C in sterile 0.9 % Hanks balanced salt solution HBSS–HEPES buffer (w/v; pH 7.4; Gibco, USA) supplemented with 1 % penicillin (v/v; 500 U/ml) and streptomycin (v/v; 500 μg/ml, Sigma-Aldrich) until use. Under sterile conditions, one end of each umbilical cord vein was clamped shut, and 0.025 % collagenase type II (w/v; Worthington Biochemicals Corporation, USA) in Puck’s saline A solution (PSA; Gibco, USA) was gently infused into the vein lumens. After the remaining open ends of the umbilical veins were clamped, they were incubated at 37 °C in 5 % CO_2_ for 20 min. The umbilical veins were then gently massaged and unclamped, and the resulting collagenase solutions were collected individually in 50 ml plastic tubes (Nunc, USA) containing 1 ml of fetal calf serum (FCS; Gibco) to inactivate the collagenase. The umbilical vein lumens were then washed twice with RPMI 1640 culture medium (Gibco). The washes were pooled, centrifuged (400 × *g*, 10 min), resuspended in complete endothelial cell growth medium (ECGM; PromoCell, Germany), plated in 25 cm^2^ plastic tissue culture flasks (Nunc), and incubated at 37 °C in 5 % CO_2_. The BUVEC monolayers were fed complete ECGM one day after isolation and then every 2–3 days thereafter. The purity of BUVEC was at least 97 % and characterized by their typical cobblestone morphology and by the incorporation assay of Dil-Ac-LDL (dioactecyltetramethyl-indocarbocyanine perchlorate acetylated low-density lipoprotein) as described elsewhere, [54]. The cells were used for the NEFA experiments after 1–6 passages in vitro.

BUVEC cultures were fed three times a week with modified ECGM culture medium: 70 % M199 (Gibco) with 30 % complete ECGM supplemented with epithelial and endothelial growth factors, heparin (all from PromoCell), 2 mM glutamine, 2 % FCS, 100 U/ml penicillin, and 100 μg/ml streptomycin sulfate (HyClone, Thermo Scientific, USA). The cells were maintained at 37 °C in a 5 % CO_2_ atmosphere.

### Intracellular calcium measurement

For the NEFA experiments, BUVEC monolayers were trypsinized with 0.25 % trypsin/EDTA (Gibco) from plastic tissue culture plates (Life Technologies, USA), and 5 × 10^6^ cells/ml were suspended and incubated for 30 min at 37 °C in HEPES-buffered saline (HBS) buffer (concentrations in mM: 150 NaCl, 5 KCl, 1 CaCl_2_, 1 MgCl_2_, 20 HEPES, 5 glucose, pH 7.4), supplemented with 10 μM Fura-2-AM and 0.02 % Pluronic F-127 (Invitrogen, Molecular Probes, USA). The unincorporated dye was removed twice with HBS buffer washes. The cells (250,000 cells/ml) were stored in HBS at 4 °C, and then prewarmed for 5 min at 37 °C. Fluorescence intensities were detected every 0.2 s at 509 nm emission and 340/380 nm dual-wavelength excitation in an LS55 spectrofluorometer (PerkinElmer Life Science, USA). The time course of FURA-2/AM in BUVECs was recorded as the ratio of the fluorescent signals without stimulus for 50 s and during exposure to each fatty acid for 100 s. The area under curve (AUC) of calcium flux was calculated for the 100 s of NEFA exposure after background subtraction. The slopes of the increase in the calcium signal caused by fatty acids were calculated with a linear regression adjusted to the first 8 s of the fluorescent signal ratio (340/380) under basal and BAPTA-AM conditions.

### NO measurement

Cells were trypsinized, as described above, seeded in a 96-well plate at a density of 20,000 cell/well, and cultured with 100 μl of ECGM-supplemented medium (PromoCell). On day 3, the cells were washed gently once with HBS and incubated for 20 min at room temperature with 5 μM DAF-FM and 0.02 % pluronic acid (Invitrogen, Molecular Probes, USA). The unincorporated dye was removed by washing once with HBS. When the cells were pretreated before fatty acid exposure, they were incubated for 15 min. The cells were then incubated with different NEFAs in the absence and presence of different treatments for 5 min at 37 °C. The DAF-FM signals were then detected with a fluorescence multiplate reader (Varioskan®, Thermo Scientific) at 480 nm excitation/520 nm emission with light detection for 800 ms.

To assess the roles of intracellular and extracellular calcium in the calcium flux and NO production in BUVECs, the cells were incubated for 30 min with 50 μM BAPTA/AM at 37 °C, then washed, and maintained in HBS. For the EGTA treatment, the cells were treated with HEPES-buffered saline without calcium, and EGTA (0.3 mM) was added just before measurement [55].

### Immunoblotting

BUVECs were lysed with a previously described method [53], and 50 μg of total protein was loaded onto a 12 % polyacrylamide gel and transferred to a nitrocellulose membrane at 100 mA for 1 h. The nitrocellulose membrane was incubated with blocking buffer for 1 h, and probed overnight at 4 °C with 0.001 μg/μl anti-human-GPR40 antiserum prepared in a rabbit (Abcam, USA). The membrane was washed three times in Tris-buffered saline and then exposed to 1:5000 anti-rabbit-IgG antibody conjugated with horseradish peroxidase (Cell Signaling) for 1 h at room temperature. Blocked antibody solutions were prepared with 1 % fat-free milk phosphate-buffered saline (PBS)–Tween 0.1 % (PBST), and signals were detected with an enhanced chemiluminescence system.

### Indirect immunofluorescence

Cells were grown on 12 mm glass coverslips, fixed with 4 % paraformaldehyde (Merck), permeabilized with 100 μM digitonin (Sigma-Aldrich). They were incubated with 0.010 μg/μl anti-GPR40 antiserum (Abcam, USA) overnight at room temperature, and then with 0.4 μg/μl Alexa-Fluor-488-conjugated anti-rabbit IgG antibody (Calbiochem, USA) for 1 h at room temperature. The anti-GPR40 antiserum was raised against the human epitope, but this particular peptide region is conserved in bovine species, as the predictive sequence indicates [13]. This antibody has been used successfully in our laboratory, as described elsewhere [14]. The blocking and antibody solutions were prepared with 1 % BSA–PBST (Sigma-Aldrich). Cell nuclei were stained with 0.1 μg/μl propidium iodide (Invitrogen, Molecular Probes, USA) diluted in PBS. The images were obtained on a Fluoview SV1000® confocal microscope (Olympus) (Centre of Scientific Studies, Valdivia, Chile) equipped with a digital camera.

### Cell death assay

Cells were seeded in 96-well plates at 20,000 cells/ml, and on day 3, were washed gently with HBS before exposure to NEFAs for 15 min at 37 °C. As the positive control for cell death, cells were treated with 0.2 % Triton X-100 diluted in HBS at 37 °C for 15 min. BUVECs were treated with different single NEFAs (300 μM LA, PA, OA, MA, or SA), Triton X-100, or 1 % vehicle (DMSO or ethanol). The cells were then incubated with 5 μM propidium iodide (Molecular Probes, Invitrogen) in HBS for 15 min. The propidium iodide signals were detected with a fluorescence multiplate reader (Varioskan®, Thermo Scientific) at 530 nm excitation/620 nm emission.

### NEFA effects on adhesion molecule expression in BUVECs

To determine the effects of NEFAs on the expression of adhesion molecules, confluent BUVEC monolayers were treated with 300 μM MA or vehicle (0.1 % ethanol), or 300 μM OA, 300 μM LA, or vehicle (0.1 % DMSO) for 3 h and their total RNA was then isolated. In another set of experiments, BUVECs were preincubated with 10 μM GW1100 or 4 μM U73122 for 30 min before LA treatment.

### RNA isolation and RT–PCR analysis

Total RNA was isolated from BUVECs with the E.Z.N.A Total RNA Kit® (Omega Bio-Tek, Norcross, GA, USA), according to the manufacturer’s instructions. The samples were treated with ribonuclease-free deoxyribonuclease I (Turbo DNA-free Kit, Ambion, UK) to remove any genomic DNA, and the RNA was quantified with a NanoDrop 2000 spectrophotometer (Thermo Fisher Scientific). For cDNA synthesis, 200 ng of RNA was reverse transcribed in the presence or absence of M-MLV reverse transcriptase (Promega). The PCR reaction was performed with Maxima SYBR Green/ROX qPCR Master Mix and 1 μL of cDNA. The primers for ICAM-1 and IL-8 used in this study were designed previously [15, 53]. SDHA cDNA was also amplified as the housekeeping control [56].

### Statistical analysis

The results are illustrated in bar graphs as the means ± SEM of at least four independent experiments. Student’s *t* test or one-way analysis of variance and Dunnett’s multiple comparison test were performed with a significance level of 5 %, using GraphPad Prism for Mac OS X (ver. 5.0; GraphPad Software, USA).

## Additional files

Additional file 1:
**NEFA treatment did not affect BUVEC cell viability.** Cells were incubated for 15 min in the presence of HBS alone (basal), 0.3 mM EGTA (in HBS), solvent (DMSO or ethanol), 300 μM of each fatty acid or 300 μM fatty acid plus EGTA. In other experimental set, cells also were incubated for 30 min with 50 μM BAPTA-AM in HBS and then exposed to each fatty acid for 15 min. Afterwards, the cells were exposed to propidium iodide in HBS for 15 min. The signals of the incorporated propidium iodide were detected with a fluorescence multiplate reader in arbitrary units of fluorescence. Each bar represents the mean ± SEM of at least three independent experiments. Myristic acid (MA), palmitic acid (PA), stearic acid (SA), linoleic acid (LA), oleic acid (OA). ***p* < 0.05 compared with basal. (PPT 128 kb) Additional file 2:
**Effects of BAPTA and EGTA treatments on the slope of the Fura-2/AM signal in cells treated with myristic acid (MA) palmitic acid (PA), stearic acid (SA), linoleic acid (LA), or oleic acid (OA).** Data are means ± standard deviations; NS: not significant. (DOC 29 kb) Additional file 3:
**Effect of GW110 on the increase in the area under curve of Fura-2/AM, induced with myristic acid (MA) palmitic acid (PA), stearic acid (SA), linoleic acid (LA), or oleic acid (OA).** Data are means ± standard deviations; NS: not significant. (DOC 33 kb)

## Abbreviations

BAPTA-AM: 1,2-Bis(2-aminophenoxy)ethane-N,N,N',N'-tetraacetic acid tetrakis(acetoxymethyl ester)
BSA: bovine serum albumin
BUVEC: bovine umbilical vein endothelial cells
DMSO: dimethyl sulfoxide
EGTA: ethylene glycol-bis-(2-aminoethyl)-N,N,N', N'- tetraacetic acid
eNOS: endothelial nitric oxide synthase
ETOH: ethanol
FFAR1: free fatty acid receptor 1
GPR40: G protein-coupled receptor 40
HBS: HEPES buffered saline
HBSS: Hank’s balanced salt solution
ICAM-1: intercellular adhesion molecule 1
IL-8: interleukin 8
LA: linoleic acid
LTCC: L-type of calcium channels
MA: myristic acid
NEFA: non-esterified fatty acid
NO: nitric oxide
OA: oleic acid
PA: palmitic acid
PTX: Pertuxis toxin
SA: stearic acid
VCAM-1: vascular cell adhesion molecule 1
