# Supplementary figures and images for: Differential intracellular calcium influx, nitric oxide production, ICAM-1 and IL8 expression in primary bovine endothelial cells exposed to nonesterified fatty acids
Source: BMC Vet Res. 2016 Feb 25;12:38. doi: 10.1186/s12917-016-0654-3 (PMC4766702; doi:10.1186/s12917-016-0654-3)

## Slide 1
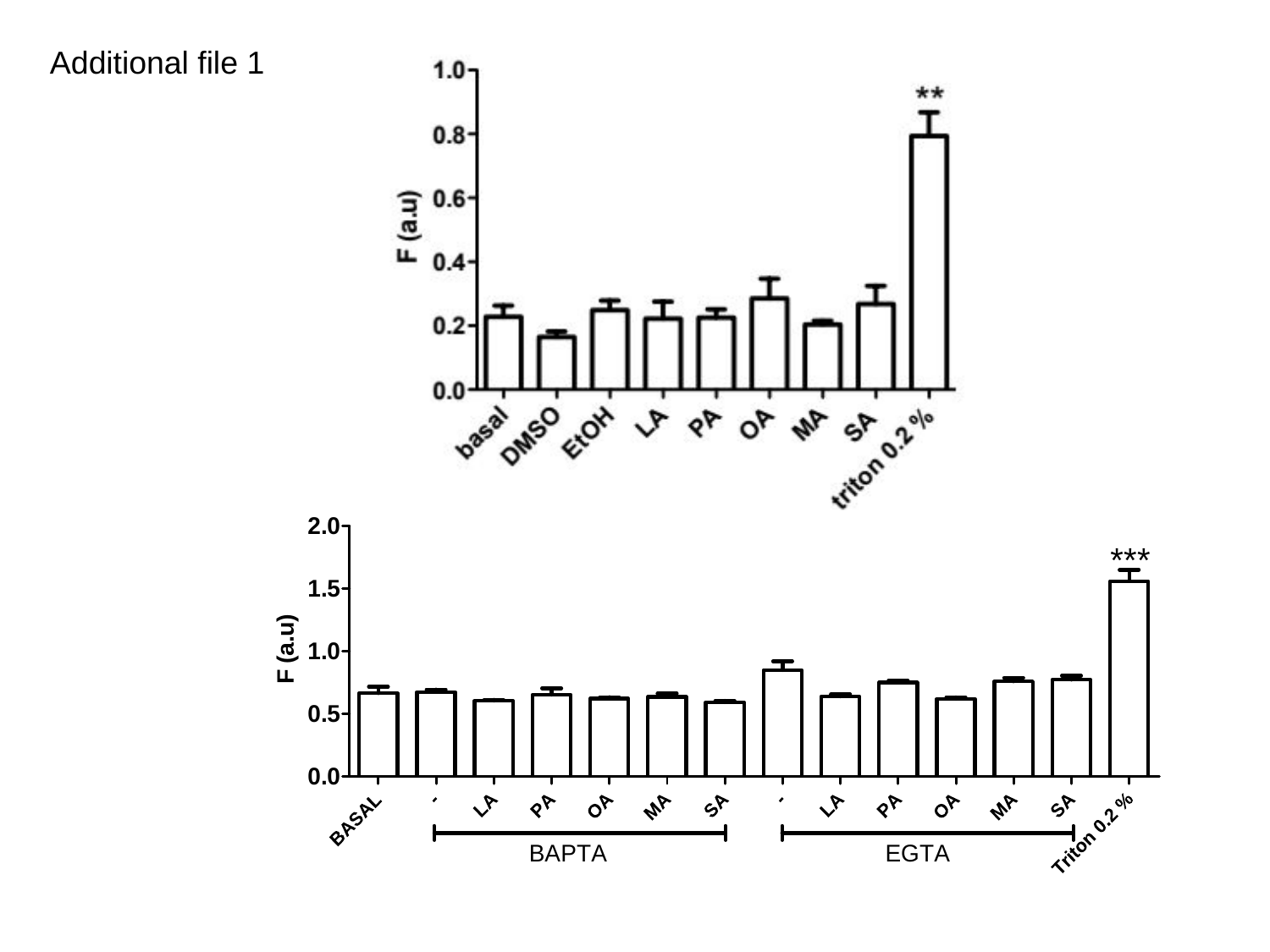

Additional file 1

Supplement: Additional file 1: — NEFA treatment did not affect BUVEC cell viability. Cells were incubated for 15 min in the presence of HBS alone (basal), 0.3 mM EGTA (in HBS), solvent (DMSO or ethanol), 300 μM of each fatty acid or 300 μM fatty acid plus EGTA. In other experimental set, cells also were incubated for 30 min with 50 μM BAPTA-AM in HBS and then exposed to each fatty acid for 15 min. Afterwards, the cells were exposed to propidium iodide in HBS for 15 min. The signals of the incorporated propidium iodide were detected with a fluorescence multiplate reader in arbitrary units of fluorescence. Each bar represents the mean ± SEM of at least three independent experiments. Myristic acid (MA), palmitic acid (PA), stearic acid (SA), linoleic acid (LA), oleic acid (OA). **p < 0.05 compared with basal. (PPT 128 kb) [file 12917_2016_654_MOESM1_ESM.ppt]
