# Supplementary material for: Differential intracellular calcium influx, nitric oxide production, ICAM-1 and IL8 expression in primary bovine endothelial cells exposed to nonesterified fatty acids
Source: BMC Vet Res. 2016 Feb 25;12:38. doi: 10.1186/s12917-016-0654-3 (PMC4766702; doi:10.1186/s12917-016-0654-3)
Supplement: Additional file 2: — Effects of BAPTA and EGTA treatments on the slope of the Fura-2/AM signal in cells treated with myristic acid (MA) palmitic acid (PA), stearic acid (SA), linoleic acid (LA), or oleic acid (OA). Data are means ± standard deviations; NS: not significant. (DOC 29 kb) [file 12917_2016_654_MOESM2_ESM.doc]

Additional file 2

| NEFA | BASAL | N | BAPTA | N | EGTA | BASAL vs BAPTA | BASAL vs EGTA |
| --- | --- | --- | --- | --- | --- | --- | --- |
| MA | 0.106 ± 0.029 | 4 | 0.049 ± 0.006 | 5 | 0.017 ± 0.001 | < 0.05 | < 0.05 |
| PA | 0.095 ± 0.024 | 3 | 0.022 ± 0.003 | 4 | 0.006 ± 0.002 | < 0.05 | < 0.05 |
| SA | 0.083 ± 0.017 | 3 | 0.025 ± 0.006 | 3 | 0.005 ± 0.002 | < 0.01 | < 0.01 |
| LA | 0.022 ± 0.016 | 5 | 0.013 ± 0.006 | 7 | 0.017 ± 0.012 | NS | NS |
| OA | 0.137 ± 0.025 | 3 | 0.033 ± 0.005 | 3 | 0.008 ± 0.003 | < 0.05 | < 0.01 |
