# Supplementary material for: Differential intracellular calcium influx, nitric oxide production, ICAM-1 and IL8 expression in primary bovine endothelial cells exposed to nonesterified fatty acids
Source: BMC Vet Res. 2016 Feb 25;12:38. doi: 10.1186/s12917-016-0654-3 (PMC4766702; doi:10.1186/s12917-016-0654-3)
Supplement: Additional file 3: — Effect of GW110 on the increase in the area under curve of Fura-2/AM, induced with myristic acid (MA) palmitic acid (PA), stearic acid (SA), linoleic acid (LA), or oleic acid (OA). Data are means ± standard deviations; NS: not significant. (DOC 33 kb) [file 12917_2016_654_MOESM3_ESM.doc]

Additional file 3

|  | BASAL | N | GW1100 | N | P |
| --- | --- | --- | --- | --- | --- |
| MA | 96.74 ± 20.92 | 5 | 34.44 ± 8.332 | 5 | < 0.05 |
| PA | 116.3 ± 9.235 | 3 | 40.54 ± 22.81 | 3 | < 0.05 |
| SA | 71.05 ± 16.27 | 4 | 26.14 ± 9.146 | 3 | < 0.05 |
| LA | 78.93 ± 8.613 | 5 | 85.87 ± 19.22 | 3 | NS |
| OA | 107.6 ± 8.182 | 3 | 95.76 ± 5.328 | 4 | NS |
